# Exploring the relationship between metabolism and immune microenvironment in breast cancer bone metastasis based on metabolic pathways

**DOI:** 10.1371/journal.pone.0341270

**Published:** 2026-01-29

**Authors:** Changyou Yang, Zhaofeng Li, Guojian Li, Houlin Mi, Yi Qin

**Affiliations:** Zhuhai Clinical Medical College of Jinan University (Zhuhai People’s Hospital, The Affiliated Hospital of Beijing Institute of Technology), Zhuhai, Guangdong, China; Hong Kong Metropolitan University, HONG KONG

## Abstract

**Background:**

Bone metastasis is a significant contributor to mortality in patients with advanced breast cancer. Its progression is deeply intertwined with tumor metabolic reprogramming and the remodeling of the immune microenvironment. However, the dynamic interplay between metabolic pathways and immune regulation remains incompletely elucidated.

**Methods:**

In this study, leveraging RNA-seq data and clinical information from breast cancer bone metastasis (BCBM) patients sourced from the GEO database, integrated bioinformatic analyses were employed to determine the activity of metabolic pathways significantly associated with prognosis. Metabolism-related genes were identified, and different metabolism-related gene clusters (MRGs) were subsequently identified by unsupervised clustering. Furthermore, a risk model was constructed based on hub prognostic genes, and differences in immune cell infiltration and drug sensitivity were compared between different subgroups. Finally, through single-cell RNA sequencing (scRNA-seq) analysis, we elucidated cellular heterogeneity and cell-cell communication within the tumor microenvironment (TME).

**Results:**

This study identified three metabolic pathways (Amino Acid, Cofactor/Vitamin, and Secondary Metabolite) significantly associated with patient prognosis. Two metabolic pathway-related subtypes (C1 and C2) were defined, which exhibited differing prognostic outcomes. Concurrently, MRG1–3 were also identified, and there were significant differences in prognosis and immune infiltration levels between the three clusters, with MRG2 having a significantly better prognosis than MRG1 and MRG3. In addition, metabolism-related risk models based on risk scores were developed. The risk model had strong prognostic predictive ability. Subsequently, scRNA-seq analysis revealed that ALDH1A1 and macrophages may play a key role in BCBM.

**Conclusion:**

This study reveals the prognostic metabolic pathways and important prognostic target genes in BCBM from the perspective of metabolism-immunity interaction. MRGs can well distinguish the prognosis of different patients, and metabolism-related risk modeling can be used as a good prognostic predictor, which provides valuable insights into the “metabolic-immune” perspective of treatment.

## Introduction

Breast cancer is the most prevalent malignancy among women, surpassing lung cancer as the most frequently diagnosed cancer type globally. Bone represents the most common metastatic site for breast cancer, with bone metastasis occurring in approximately 67% of patients with advanced disease [1,2]. According to statistics from the American Cancer Society, patients with BCBM exhibit a substantially reduced survival rate compared to those with primary breast cancer, with a reported 3-year survival rate of merely 50.5% [3,4]. Furthermore, bone metastases precipitate skeletal-related events, such as pathological fractures and hypercalcemia, which severely compromise patients’ quality of life and indirectly contribute to reduced overall survival(OS) [5,6]. Therefore, it is imperative to identify novel prognostic biomarkers and construct robust predictive models based on patient heterogeneity characteristics, to guide individualized therapeutic strategies.

The bone marrow, a specialized immune organ, has a unique immune cell composition compared to peripheral circulation and other tissues. Unlike the peripheral blood where T cells comprise 45−75% of lymphocytes, bone marrow niches contain <5% T cells among total immune cells [7,8]. This distribution arises from an intrinsically immunosuppressive microenvironment enriched with immature or functionally suppressive immune cells, including functionally active regulatory T cells (Tregs) and myeloid-derived suppressor cells (MDSCs) [9]. Preclinical studies reveal that immune cells within the bone metastatic niche drive tumor progression through multifaceted mechanisms. Tregs, a critical immunosuppressive subset of CD4 ⁺ T cells, inhibit CD8 ⁺ T and Th1 cell functionality in bone metastatic cancers by enhancing IL-10, IL-35, and TGF-β signaling [10].Concurrently, MDSCs suppress T-cell activity through IL-2 receptor blockade and ROS/NO production while inhibiting NK cells via TGF-β [11, 12]. In the bone microenvironment, this immunosuppression is further influenced by bone tissue-specific cells(osteoblasts/osteoclasts). There is a bidirectional interaction between the immune system and bone cells [12]. Tumor-derived PTHrP, IL-7, and IL-8 recruit T cells that secrete TNF-α and RANKL to potentiate osteoclast maturation and bone resorption [13]. Concurrently, osteoblasts undergo metabolic reprogramming (e.g., aerobic glycolysis) to generate lactate-enriched niches promoting Treg-mediated immunosuppression, with CTLA-4-dependent cell contact further modulating osteoclast differentiation [14,15]. These interactions establish a “cold tumor” phenotype in bone metastases, fundamentally differing from primary tumors or other metastatic sites [16,17]. Immune checkpoint inhibitors being mainstays of cancer immunotherapy, but their efficacy against bone metastases remains limited and clinically contentious.

Metabolic reprogramming refers to the adjustment of cellular metabolic pathways in response to specific physiological or pathological conditions, enabling cells to adapt to environmental changes and support growth and survival [18]. Within the TME, tumor cells utilize metabolic reprogramming to engage in crosstalk with neighboring non-tumor cells – particularly immune cells – thereby attenuating antigen presentation and recognition capabilities and evading immune-targeted therapies [19]. For instance, bone-metastatic breast cancer cells exhibit elevated expression of phosphoglycerate dehydrogenase—the rate-limiting enzyme in serine biosynthesis, which fuels L-serine metabolism to drive osteoclastogenesis and tumor proliferation, thereby directly promoting osteolytic destruction [20,21]. For example, research has demonstrated that excessive glutamate derived from tumor glutaminolysis upregulates metabotropic glutamate receptor 1 expression in Tregs, thereby enhancing their glutamate uptake and metabolic utilization to drive Tregs proliferation and tumor infiltration [22]. Concurrently, glycolytic activation in tumor cells promotes granulocyte colony-stimulating factor overexpression, recruiting MDSCs to suppress antitumor immunity [23,24]. In addition, due to molecular characteristics, gene expression, and microenvironment heterogeneity between primary and metastatic sites, metastatic sites exhibit unique metabolic characteristics, rendering conventional “metabolic-immune” targets ineffective [25]. The research by Feng et al. demonstrates that within the same type of tumor, patients can be divided into subgroups suitable for different treatment methods based on different metabolic characteristics [26]. Thus, delineating metastasis-specific metabolic signatures in BCBM, combined with decoding the resultant immunomodulatory landscape, provides a mechanistic foundation for precision therapeutics [27].

This study, based on publicly available database data, aims to comprehensively characterize prognostic metabolic pathways in BCBM, stratify tumor subtypes by metabolic signatures, and investigate their underlying pathophysiological features through integrated immune analyses. Subsequently, we will establish a prognostic risk model and leverage scRNA-seq to elucidate differential expression patterns of outcome-associated genes and metabolic pathways within the TME, thereby providing insights for developing “metabolic-immune” therapies targeting BCBM.

## Methods

### Data collection and processing

RNA-seq data and clinical information from 33 BCBM bone tissue samples from the GES175692 were obtained from the GEO database (https://www.ncbi.nlm.nih.gov/geo/). Additionally, 84 human metabolic subsets were retrieved and downloaded from the KEGG database (https://www.genome.jp/kegg/) and integrated into eight major classical metabolic pathways.

### Pathway enrichment analysis

To quantify the activity of the eight major metabolic pathways in each sample, gene set variation analysis was performed using the R package “GSVA”(v1.50.5) plugin to calculate the enrichment scores for each pathway in individual samples [28]. Subsequently, Kaplan-Meier survival analysis evaluated the association between metabolic pathway activity and OS in BCBM. In addition, the R package “Survminer”(v0.50.0) was used to determine the optimal cut-off point for each metabolic pathway using “cut-point” values to distinguish between high and low groups.

### Identification of metabolic subtypes and analysis of immune infiltration

To systematically characterize metabolic patterns in BCBM, unsupervised consensus clustering was performed using the R package “ConsensusClusterPlus”(v1.70.0) to identify potential metabolic subtypes. The 80% of the total sample involved 1,000 iterations across k-values (2–10), with optimal cluster determination via consensus cumulative distribution functions and heatmap analysis [29,30]. For comprehensive immune microenvironment profiling, we used the ESTIMATE and CIBERSORT methods. The ESTIMATE algorithm is a method for inferring the overall level of immune infiltration in tumor tissue based on gene expression data [31]. In this study, we used this algorithm to infer the ImmuneScore, StromalScore, and ESTIMATEScore of BCBM [32]. The “CIBERSORT” analysis quantified 22 human immune cell subset abundances in BCBM [33]. Pairwise immune cell correlations were computed using Pearson coefficients, visualized via ggplot2(v3.5.1) correlation heatmaps.

### Identification of metabolism-related genes

Differential gene expression analysis between metabolic subtypes was performed using the R package “limma” (v3.56.2). Genes meeting thresholds of p-value < 0.05 and |log2(fold change) | > 1 were defined as metabolism-related. Gene Ontology (GO) and Kyoto Encyclopedia of Genes and Genomes (KEGG) pathway enrichment analyses were performed on the metabolism-related genes to elucidate the biological processes and signaling pathways underpinning these genes. In addition, due to cohort size limitations, prognostic gene selection employed Elastic Net regularization (alpha = 0.5) – a linear regression model integrating L1 (Lasso) and L2 (Ridge) penalties to mitigate overfitting [34]. Based on the prognostic genes, unsupervised cluster analysis was used to identify potential MRGs, while analyzing differences in survival outcomes and immune infiltration levels between different MRGs.

### Machine learning screening center for prognostic genes

Hub prognostic genes were identified by applying six machine learning algorithms—Random Forest (RF), Support Vector Machine (SVM), Least Absolute Shrinkage and Selection Operator (LASSO), Neural Network (NNET), and Gradient Boosting (XGBoost). Divide the samples into training and validation sets at a 7:3 ratio. Take the intersection of the top 50% genes ranked by each algorithm as the core prognostic genes. Simultaneously, And the predictive accuracy of each model was evaluated using receiver operating characteristic (ROC) curves. Subsequently, Univariate Cox regression was used to assess the correlation between clinical parameters and patient prognosis, and the optimal coefficients in the elastic net model were used to construct a risk prognosis model, calculated as:


risk score=∑Kj ×Expi

where *kj* denotes the coefficient for each gene in the model and *Expi* represents its expression level in the sample. Subsequently, the median risk score is used as the cutoff point (with values below the median assigned to the low-risk group), dividing patients into high-risk and low-risk groups. To further assess the predictive accuracy of the risk model, the ROC curve analysis was performed using the “timeROC” (0.4) package [35].

### Drug sensitivity analysis

Drug sensitivity prediction was performed using the R package “oncoPredict”(v.1.2) [36]. This analysis utilized the half-maximal inhibitory concentration (IC50) values for 198 compounds from the Genomics of Drug Sensitivity in Cancer database (https://www.cancerrxgene.org/). Using the IC50 values corresponding to the expression profiles of the cell lines as the training set and the patients’ gene expression data as the test set, linear regression analysis was performed to predict individual responses to different drugs and identify novel treatment candidates for different BCBM subtypes.

### scRNA-seq analysis

BCBM-related scRNA-seq data were obtained from the GEO database, specifically the dataset GSE169246 based on the platform GPL20795, which includes six samples (GSM5188377, GSM5188384, GSM5188401, GSM5188411, GSM5188417, GSM5585281), two samples (GSM6870693, GSM6870694) from dataset GSE190772, and one sample (GSM8162626) from dataset GSE262288, all from the same platform GPL24676. All sample data were integrated and processed using the R package “Seurat” (v.4.3.3). The IntegrateData function was used to merge single-cell data. Exclude cells with gene expression levels below 200, mitochondrial gene proportions exceeding 5%, and hemoglobin gene expression exceeding 1%.Then, batch effect correction was performed using the R package “Harmony”(v1.2.3), and after the data underwent dimensionality reduction (PCA method),normalization (SCTransform function) and cluster using the FindClusters function, tSNE and UMAP were used for visualization. Finally, cell annotation was performed on different clusters using cell marker genes reported in the literature and analysis from the CellMarker2 website (http://117.50.127.228/CellMarker/).

### Cell–cell communication analysis

CellChat (v.1.6.1), an R-based computational platform, employs systematically curated ligand-receptor interaction databases to infer intercellular communication networks from single-cell transcriptomic data [37]. Using this framework, we interrogated cell-cell communication between subpopulations exhibiting ALDH1A1-differentially expressed subgroups. Parallelly, use the R package “AUCell”(1.28.0) to analyze the metabolic pathway activity of each cell in single-cell data and analyze the interactions between cells with high versus low prognostic metabolic pathway activity to systematically explain the complex relationship between metabolism and immunity in the TME [38].

### Pseudo-time analysis

Pseudo-time analysis, also known as cell trajectory analysis, enables the inference of cellular differentiation trajectories or the evolutionary processes of cell subtypes during development by examining the temporal changes in gene expression levels across different cell subpopulations. The R package Monocle3 (v1.4.26) was employed to reconstruct the differentiation trajectories of macrophage subpopulations and estimate pseudo-time orderings.

### Statistical analysis

Intergroup differences were assessed using unpaired Student’s t-tests or Wilcoxon rank-sum tests for two-group comparisons, while Kruskal-Wallis tests were employed for multiple-group analyses. The correlation between two groups was calculated using Sperman’s correlation analysis. Statistical significance was defined as two-tailed p < 0.05, with all computations performed in R (v4.3.3).

## Results

### Identification of key metabolic pathways in BCBM

**Fig 1:**
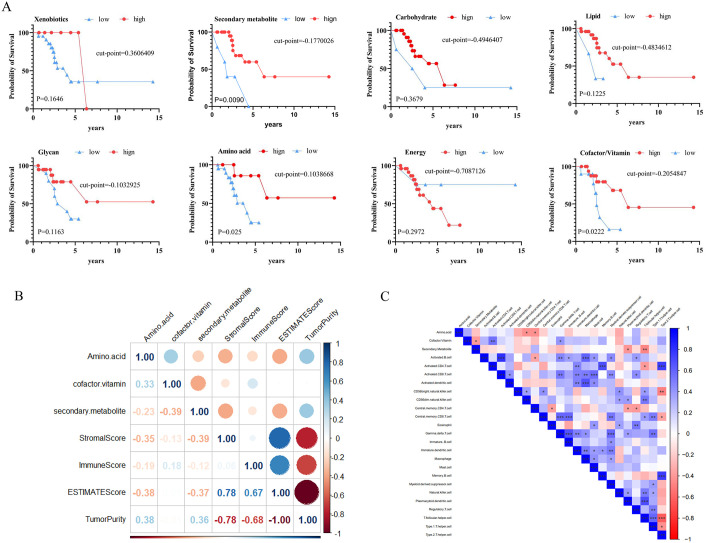
Identification of key metabolic pathways in BCBM. (A) OS differences between high and low activity groups of metabolic pathways in the Kaplan-Meier curves. Red indicates high-activity groups, blue indicates low-activity groups. (B) Correlation between 3 key metabolic pathways and immune infiltration scores. (C) Correlation between 3 key metabolic pathways and abundances of 28 immune cell types. Correlation coefficients were calculated using Spearman’s analysis. Red denotes negative correlation; blue denotes positive correlation. *p < 0.05, **p < 0.01, ***p < 0.001.

Metabolic reprogramming in tumor cells significantly influences clinical outcomes. By quantifying activity levels of eight metabolic pathways, Kaplan-Meier analysis identified three key pathways prognostic for BCBM: Amino acid metabolism, Cofactor/Vitamin metabolism, and Secondary metabolite metabolism. High levels of Amino acid (*p* = 0.025), Cofactor/Vitamin (*p* = 0.0222), and Secondary metabolite (*p* = 0.009) were associated with longer OS in BCBM, with statistical significance (Fig 1A). The Spearman correlation heatmap shows that these metabolic pathways are significantly associated with immune markers in the TME. Amino acid metabolism is negatively correlated with StromalScore (r = −0.35) and positively correlated with TumorPurity (r = 0.38). Additionally, synergistic and antagonistic relationships were observed among different metabolic pathways: Positive correlation between amino acid and cofactor/vitamin metabolism (r = 0.33), but negative correlation with secondary metabolite metabolism (r = −0.39), revealing the complex metabolic characteristics of BCBM (Fig 1B). As shown in Fig 1C, immune infiltration analysis demonstrated that elevated Amino acid activity was significantly positively correlated with immune cells such as central memory T cells, while heightened cofactor/vitamin metabolism showed a potential positive trend with activated CD4^+^ T cell infiltration.

### Identification of metabolism-related subtypes

**Fig 2:**
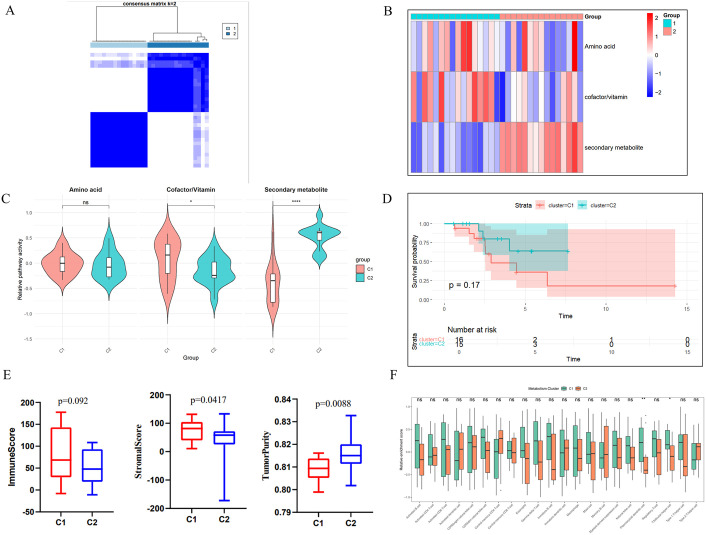
Metabolic pathway-related subtypes. (A) Consensus heatmap of metabolic pathway clustering. (B-C) Heatmap and differential expression patterns of key metabolic pathways between C1 and C2. (D) Kaplan–Meier curves depict the OS differences between C1 and C2. (E) Differences of ImmuneScore, StromalScore, and TumorPurity between C1 and C2. (F) Differential abundances of 22 immune cell between C1 and C2. *p < 0.05, **p < 0.01, ***p < 0.001.

To systematically delineate metabolic heterogeneity in BCBM, unsupervised clustering was performed based on three key pathways. The results are shown in Fig 2A, patients were clearly stratified into two distinct metabolic pathway-related subtypes (C1 and C2). C1 exhibited significantly elevated overall metabolic activity than C2 (*p* < 0.01), characterized by higher Cofactor/Vitamin (*p* < 0.05) and Amino acid metabolic activity, while C2 exhibits higher secondary metabolite activity(*p* < 0.001) (Fig 2B-C). Kaplan-Meier survival analysis showed that C1 patients had relatively better OS, but this difference did not reach statistical significance due to limited sample size (Fig 2D). In addition, we explored the relationship between metabolic pathway-related clusters and the TME of BCBM. Using the ESTIMAT algorithm to quantify TME features, the results showed that that the ImmuneScore of C1 was higher than that of C2, but the difference was not statistically significant. However, the StromalScore of C1 was higher than that of C2, while the TumorPurity was lower than that of C2, with both differences being statistically significant (*p* < 0.05) (Fig 2E-F). Crucially, Immune infiltration analysis further revealed significant differences between the two groups in terms of γδT cell and helper T cell infiltration levels (*p* < 0.05). These findings establish that different metabolic subtypes exhibit different immune states in BCBM. This prognosis-associated metabolic-immune coupling provides a novel framework for targeted therapeutic development.

### Identification of metabolic-related gene clusters

**Fig 3:**
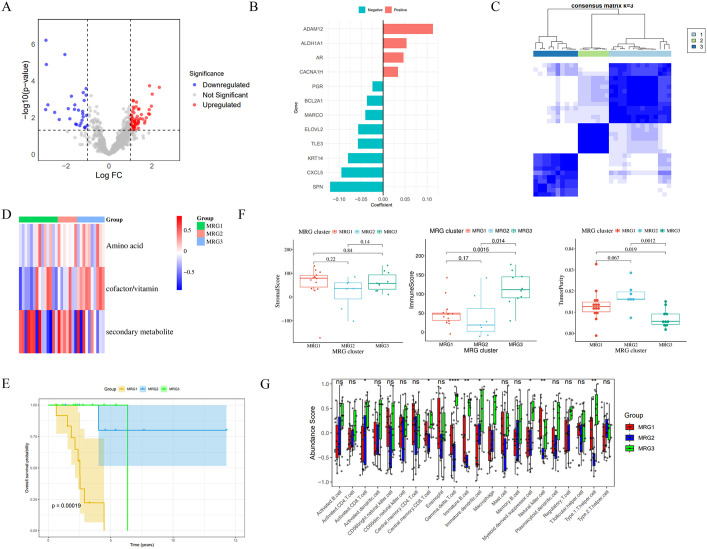
Identification of MRGs. (A) Volcano plot of differentially expressed genes between C1 and C2. (B) Prognostic gene selection via elastic net modeling. (C) Consensus heatmap of MRGs. (D) Metabolic pathway activity heatmap across MRG1-3. (E) OS stratification by MRGs. (F) Differential ImmuneScore, StromalScore, and TumorPurity among MRGs. G: Varied abundances of 22 immune cell types across MRGs. *p < 0.05, **p < 0.01, ***p < 0.001.

To further characterize and understand the biological features of MRGs and identify more effective classifications,108 differentially expressed genes (DEGs) were identified between metabolic subtypes C1 and C2, among them, 73 genes were found to be overexpressed in C1, while 35 genes were found to be underexpressed relative to C2 (Fig 3A). Subsequent GO analysis indicated that DEGs were significantly enriched in biological processes such as immune response and positive regulation of phosphorylation. Correspondingly, KEGG enrichment analysis revealed that DEGs were enriched in signaling pathways such as cytokine-cytokine receptor interactions (S1 Table). Subsequently, 12 representative prognostic DEGs were identified through Elastic Net regression analysis (Fig 3B). Based on these genes, unsupervised cluster analysis was used to divide patients into three distinct: MRG1–3 (Fig 3C). Metabolic characterization indicated that MRG2 exhibited peak global metabolic activity, whereas MRG3 displayed uniquely elevated cofactor/vitamin metabolism (Fig 3D). Critically, survival analysis revealed that MRG2 had significantly better prognosis than MRG1 and MRG3, with statistically significant differences (*p* < 0.001) (Fig 3E). This indicates that MRG classification can distinguish patients into groups with significant survival differences. Furthermore, immune microenvironment profiling revealed that MRG2 concurrently demonstrated the lowest ImmuneScore and highest TumorPurity both of which were statistically significant compared to the other two groups (*p* < 0.05) (Fig 3F). In addition, the results of the CIBERSORT analysis demonstrated significant disparities in the levels of various immune cells, including CD8 T cells, macrophages, and NK cells, between MRGs (*p* < 0.05) (Fig 3G). Consequently, the MRGs classification outperforms metabolism-related subtypes in terms of prognostic stratification and unveils distinct immunophenotypic landscapes, providing a molecularly-defined framework for therapeutic targeting.

### Construction of a metabolic risk model

**Fig 4:**
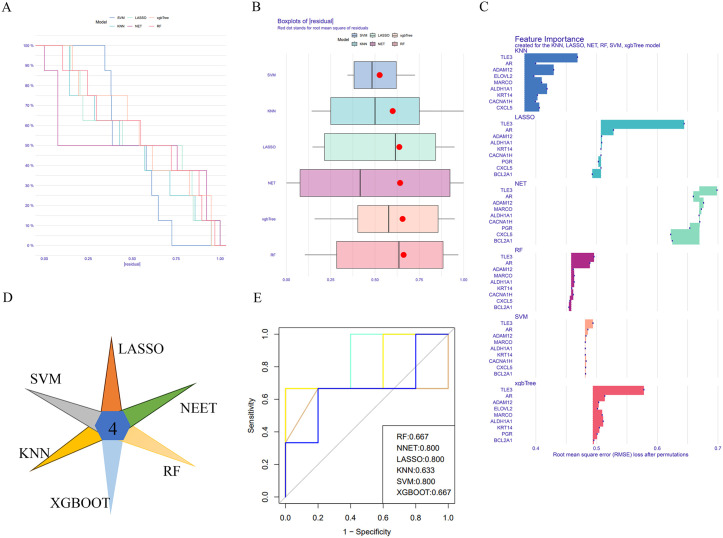
Machine learning methods identify central prognostic genes. (A-B) Reciprocal cumulative distribution curves determine model accuracy. (C) Top 10 genes among the six models. (D) Venn diagram identifies genes common to all six models. (E) ROC curve.

**Fig 5:**
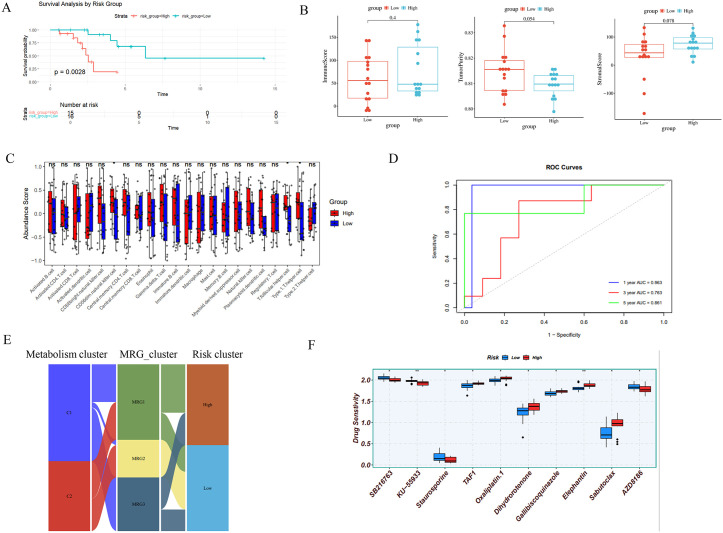
Construction of a metabolism-related risk model. (A) Kaplan-Meier curves of OS differences between high- and low-risk groups. (B) Differences in ImmuneScore, StromalScore, and TumorPurity between high- and low-risk groups. (C) Differences in the abundance of 28 immune cell types between high- and low-risk groups. (D) AUC values at 1, 3, and 5 years in the timeROC curve. (F) Drug sensitivity analysis between high- and low-risk groups. *p < 0.05, **p < 0.01, ***p < 0.001.

To identify hub prognostic genes, we integrated six machine learning approaches (RF, SVM, LASSO, NNET, XGBoost), with inverse cumulative distribution and box plots confirming the SVM model’s minimal residual values and optimal predictive performance (Fig 4A-B). Venn diagram intersection of the top 50% genes across every models identified four hub genes (ADAM12, ALDH1A1, TLE3, PGR) (Fig 4C-D). ROC curve results showed that the AUC values of each model were greater than 0.6, indicating good diagnostic capability (Fig 4E). In addition, to further construct a risk prediction model related to metabolism, we first performed a univariate analysis of the clinical information provided in the dataset and found that there were no clinical variables that significantly affected patient prognosis (S2 Table).We then selected the optimal coefficients from the Elastic Net model to construct the risk model, calculating the risk score using the formula: Risk Score = ALDH1A1 × 0.053081 + ADAM12 × 0.113421 + TLE3× −0.05728 + PGR × −0.02411.Patients were stratified into high-risk and low-risk groups based on median risk scores. The findings indicated that patients in the low-risk group with BCBM had significantly better OS with statistical significance (*p* < 0.01) (Fig 5A). Immunological characteristics also differed between the high-risk and low-risk groups, with higher immunological scores in the low-risk group, consistent with the conclusion of better patient outcomes. However, the results did not reach statistical significance (*p* > 0.05) (Fig 5B-C). Time-dependent ROC analysis validates that the AUC values for patients at 1 year, 3 years, and 5 years are 0.963, 0.763, and 0.861, respectively, confirming that the risk score is a good predictor of OS in the cohort (Fig 5D).The cumulative distribution plot shows that most C1 patients were assigned to the high-risk group, most C2 patients were assigned to the low-risk group, and the better-prognosed MRG2 was entirely assigned to the low-risk group, reflecting a significant association among the “metabolic subtype-MRGs-risk grouping” trio (Fig 5E). Drug sensitivity analysis was conducted, which confirmed an enhanced therapeutic vulnerability to the BCL-2 inhibitor, Sabutoclax, and the kinase inhibitor, Staurosporine, in patients with a high risk (*p* < 0.05) (Fig 5F). In a consistent manner, Staurosporine exhibited selective efficacy against the C1 metabolic subtype (S3 Table), while CDK9 inhibitor CDK.9.1 and reversible proteasome inhibitor MG-132 demonstrated preferential activity in MRG3—the MRGs with poorest prognosis.

### Differential expression of ALDH1A1 in scRNA-seq

**Fig 6:**
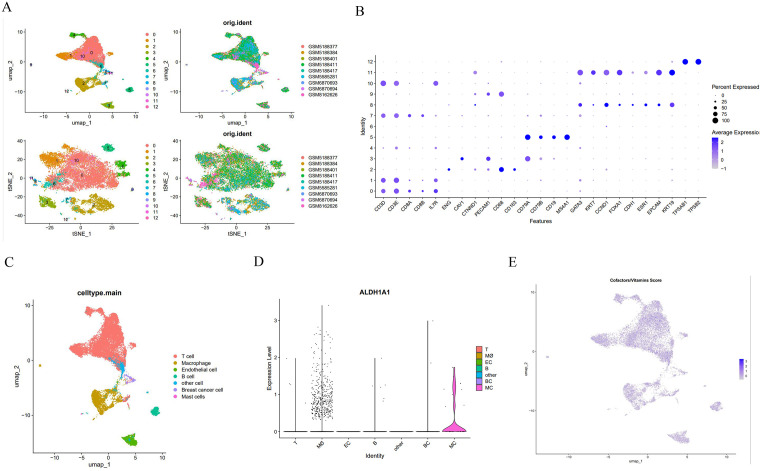
scRNA-seq analysis. (A) UMAP and t-SNE visualizations depicting sample composition and distribution. (B) Expression of signature genes across 12 cell clusters. Dot size corresponds to the proportion of cells expressing specific markers, while color indicates the average expression level of each marker. (C) UMAP plot annotated with major cell types. (D) Differential ALDH1A1 expression across annotated cell types. (E) UMAP plot of cofactor/vitamin differentially expressed in each cell type.

To delineate hub prognostic gene expression heterogeneity at scRNA-seq, seven BCBM scRNA-seq samples from GEO were integrated. Following the implementation of quality control measures, a total of 24,201 cells, which expressed 17,173 genes, were subjected to further analysis and subsequently categorized into 12 distinct populations (Fig 6A). Differential gene expression analysis revealed widespread gene expression dysregulation in different cell types in BCBM compared to controls. The cell clusters were further identified as seven cell types, including breast cancer cells, endothelial cells, T cells, B cells, macrophages, mast cells, and other cells (Fig 6B-C). Notably, ALDH1A1—a hub gene with high positive coefficient in our risk model—exhibited the most pronounced cell-type-specific expression heterogeneity among prognostic hubs, showing maximal enrichment in macrophages, suggesting that “ALDH1A1-macrophages” may play a crucial role. (Fig 6D). Complementarily, the “AUCell” results indicate that significant cofactor/vitamin metabolism activation in both malignant and immune cells (Fig 6E), while amino acid and secondary metabolite exhibited weaker differential expression.

**Fig 7:**
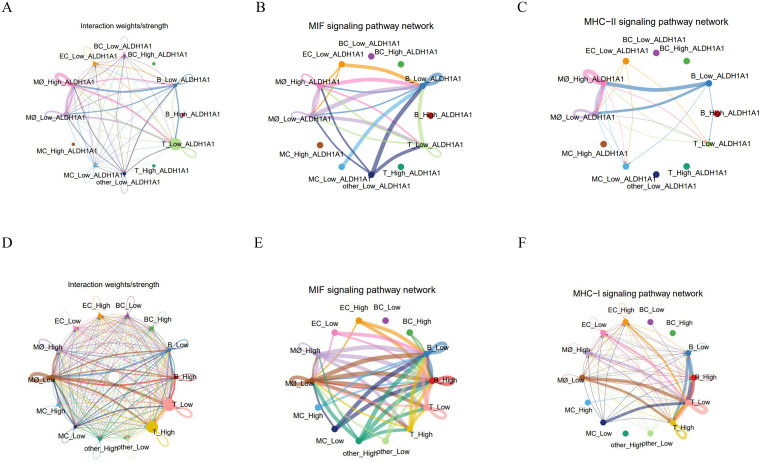
Cell-cell communication analysis. (A) Cellular interaction networks and communication strength between high- vs. low-ALDH1A1 expressing cells. (B-C) Top two signaling pathway networks in high/low ALDH1A1 comparisons. (D) Interaction networks and communication strength between high- vs. low-Cofactor/Vitamin activity cells. (E-F) Top two signaling pathway networks in high/low Cofactor/Vitamin activity comparisons.

CellChat analysis revealed that macrophage-derived signaling dominates intercellular communication networks between high- and low-ALDH1A1-expressing cells. Macrophages with differential ALDH1A1 expression functioned as primary signal senders, mediating communication through MIF (macrophage migration inhibitory factor) signaling pathways and MHC-II signaling pathways via ligand-receptor pairs “CD74-CXCR4” and “CD74-CD44” (Fig 7A-C). Complete interaction landscapes in S1 Fig. Additionally, cell-cell communication analysis of cells stratified by high/low cofactor/vitamin metabolic activity demonstrated significantly stronger communication intensity and broader signaling diversity compared to the ALDH1A1-based groups (Fig 7D-F). Remarkably, these interactions operated through identical core pathways—MIF and MHC-II signaling pathway—utilizing the same receptor-ligand complexes CD74-CXCR4 and CD74-CD44. This mechanistic convergence establishes ALDH1A1 as a critical functional component of cofactor/vitamin metabolism reprogramming in BCBM.

### Re-clustering of macrophage and pseudo-time series analysis

**Fig 8:**
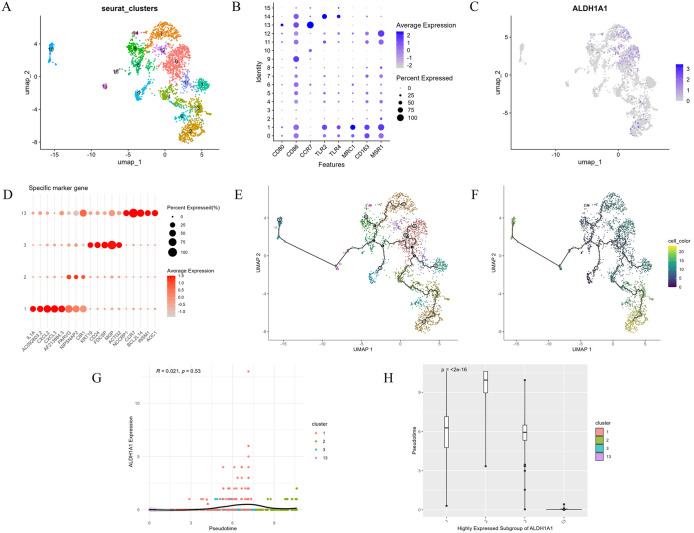
Pseudo-time analysis. (A) Macrophage Clustering. (B) M1/M2 Macrophage Markers. (C) ALDH1A1 Distribution in Macrophages. (D) Top Five Highly Variable Genes in the ALDH1A1-High Subpopulation. (E-F) Macrophage Trajectory and Pseudo-time Plots. (G) The relationship between the expression level of ALDH1A1 and pseudo-temporal sequence in the high ALDH1A1 subpopulation. (H) The distribution differences of the high-expression subpopulation of ALDH1A1 in the pseudo-temporal sequence.

To further clarify the importance of macrophages in BCBM, we extracted a subset of cells annotated as ‘macrophages’ (2,762 cells) from the entire Seurat dataset. Following re-normalization, dimensionality reduction and clustering of this subset, 15 macrophage subpopulations were identified (Fig 8A). These subpopulations were initially distinguished based on M1 markers (CD80, CD86, CCR7, TLR2 and TLR4) and M2 markers (MRC1, CD163, CD11B and MSR1). As described in the literature, M1/M2 macrophage types could not be completely separated in the single-cell data (Fig 8B) [39].Subsequently, we assessed ALDH1A1 expression across each subpopulation (Fig 8C) and identified four subpopulations that expressed ALDH1A1 highly: 1, 2, 3 and 13. Subpopulation 1 was classified as M2-type macrophages and subpopulation 13 as M1-type macrophages; subpopulations 2 and 3 remained unclassified. The top five differentially expressed genes in each highly expressed subpopulation were identified to define each unique subpopulation (Fig 8D). To investigate the expression dynamics of ALDH1A1 during macrophage differentiation, we then reconstructed the developmental trajectories of the subpopulations using Monocle3. Selecting cells with the lowest ALDH1A1 expression as the starting point allowed us to construct a pseudo-temporal differentiation pathway for macrophages (Fig 8E-F). Trajectory analysis revealed that macrophages develop along a continuous lineage of differentiation, branching into multiple directions from a state of low ALDH1A1 expression. Interestingly, ALDH1A1 expression dynamically fluctuates along the macrophage developmental trajectory, showing significant upregulation during the intermediate stage (Fig 8G-H). This suggests that ALDH1A1 may play a crucial regulatory role in macrophage differentiation by modulating cellular plasticity or metabolic reprogramming.

## Discussion

Mounting evidence indicates that metabolic reprogramming plays a central role in tumor progression and immunotherapy efficacy. Within hypoxic TME, cancer cells actively rewire metabolic pathways to establish immunosuppressive niches, compromise drug sensitivity, and accelerate the development of drug resistance, ultimately leading to treatment failure [40,41]. Although previous studies have confirmed the metabolic differences between breast cancer and normal tissues, current research on BCBM is largely limited to single metabolic pathways (such as glycolysis or amino acid metabolism) [42,43]. A salient limitation is that differences in single metabolic gene expression cannot fully reflect the dynamic remodeling of the tumor metabolic network, especially when considering the significant metabolic differences between the microenvironments of primary tumors and metastatic lesions [19]. Therefore, this study comprehensively analyzes the characteristics of BCBM at the metabolic pathway level, elucidates the complex relationship among metabolic reprogramming, immune microenvironment remodeling, and clinical prognosis, and provides a new perspective for overcoming treatment bottlenecks.

In this study, we initially examined the correlation between heightened activity in amino acid, co-factor/vitamin, and secondary metabolite pathways and improved clinical outcomes.. This finding contradicts previous research suggesting that cancer cells require more amino acids (such as glutamine) under glucose-deprived conditions [44]. However, this seemingly contradictory phenomenon highlights the complexity of metabolic reprogramming. Specifically, the effects of upregulation or downregulation of different metabolic pathways on tumor growth vary significantly, and even the same metabolic pathway may play different roles in different tumor types or microenvironments [45]. Take arginine metabolism as an example: in liver cancer cells, high levels of arginine-binding RNA-binding protein (RBM39) promote tumor growth by upregulating aspartate synthase ASNS [46]. In breast cancer, polyamines derived from arginine metabolism enhance pro-tumor macrophage polarization through thymidine DNA glycosyltransferase (TDG)-mediated DNA demethylation, thereby inhibiting CD8 + T cell activity and suppressing immunotherapy [47]. Critically, in agreement with our study, Wu et al. found that high activity of cofactor/vitamin metabolism is associated with better prognosis in osteosarcoma [48]. Additionally, due to limitations in cohort size, to further explore the clinical significance of metabolic characteristics in BCBM, we selected the Elastic Net model to screen for key prognostic genes and constructed MRGs (MRG1–3). It is worth noting that MRGs may have greater clinical significance than metabolic subtype grouping. First, there are differences in metabolic activity among MRGs, with MRG2 exhibiting higher secondary metabolite activity and MRG3 exhibiting higher cofactor/vitamin activity. Second, Kaplan-Meier survival analysis results indicate significant differences in clinical outcomes among MRGs, with MRG2 showing markedly better prognosis than MRG1 and MRG3. Paradoxically, MRG2 exhibited the lowest ImmuneScore despite optimal prognosis, which contradicts the general conclusion that higher ImmuneScore scores correlate with better prognosis in most tumors. However, based on tumor immunology theory, we speculate that this phenomenon may be associated with immune-suppressive cell infiltration [49,50]. Relevant studies have confirmed that an elevated level of Treg cell infiltration in certain breast cancers is associated with an increased risk of recurrence [51]. Consistent with the above findings, subsequent single-cell data also confirmed the central role of macrophages in the bone metastasis microenvironment. Collectively, MRG-related clusters integrate metabolic reprogramming with immune-suppressive microenvironment interactions and better reflect tumor prognosis, making them potential prognostic prediction methods. Subsequently, leveraging six machine learning algorithms, we identified four hub genes (ADAM12, ALDH1A1, TLE3, PGR) and constructed a prognostic risk model. Elevated risk scores correlated directly with poorer survival outcomes—consistent with established evidence that risk stratification optimizes therapeutic decision-making across malignancies [50]. Functional enrichment and immune deconvolution analyses further revealed intricate metabolic-immune disparities between high- and low-risk groups. Sankey diagram visualization delineated the hierarchical relationship among metabolic subtypes, MRG clusters, and risk stratification, demonstrating that low-risk patients predominantly comprised prognostically favorable MRG2 and C2 subtypes. This alignment confirms that metabolically-defined subtyping enables robust discrimination of risk groups, providing a biological rationale for clinical translation.

scRNA-seq has revolutionized our understanding of TME and anti-tumor immunity. Our analysis of scRNA-seq data revealed analyze the expression patterns of hub genes such as ADAM12, ALDH1A1, TLE3, and PGR across different cell types. Notably, significant differences in ALDH1A1 expression among various cell populations, and extensive cellular interactions were observed between cells with high and low ALDH1A1 expression, particularly in macrophages. This suggests that ALDH1A1 acts as a bridge between macrophages and TME, potentially representing a therapeutic target for cancer treatment. This aligns with emerging strategies targeting ALDH^high^ cancer stem cells (CSCs); for instance, Research has shown that relevant scholars have constructed M2-macrophage microvesicle-inspired nanovehicles of cabazitaxel (CTX) (M-CFN) to co-localize the specific marker ALDH1A1 on CSC and deeply eliminate ALDH^high^ CSC [52]. Aldehyde dehydrogenase (ALDH) is a NAD(P)⁺-dependent multifunctional protein superfamily, with ALDH1A1 being one of its primary members. [53]. Critically, in recent years, high ALDH activity has been identified as one of the hallmark features of various CSCs [54]. Extensive clinical and basic research has shown that the overexpression of multiple ALDH family members is associated with the progression, treatment resistance, and poor prognosis of various human malignant tumors, including breast cancer [53,55]. Studies have shown that ALDH1A1-high cells are enriched in triple-negative breast cancer, where they activate the TAK1-NFκB pathway to increase GM-CSF secretion, recruit MDSCs to inhibit CD8^+^ T cell immunity, and promote immune escape [56]. Concordantly, Giordano et al. noted that high immune expression of ALDH1A1 is significantly associated with poor breast cancer-specific survival (P < 0.001), particularly in luminal and triple-negative breast cancer subtypes [57]. Finally, to investigate the relationship between ALDH1A1 and macrophages in depth, we performed re-clustering of macrophage populations and employed pseudo-time analysis to elucidate the pseudo-time differentiation pathways of macrophages and the expression dynamics of ALDH1A1. Results revealed that ALDH1A1 expression dynamically fluctuates along the macrophage developmental trajectory, exhibiting significant upregulation during intermediate stages. This suggests ALDH1A1 may play a crucial regulatory role in macrophage differentiation by modulating cellular plasticity or metabolic reprogramming.

However, this study has several inherent limitations. Primarily, our data were obtained from public databases and analyzed using bioinformatics methods, lacking direct validation through in vivo and in vitro experiments. Second, due to the small sample size and limited available datasets, the risk model was evaluated solely through bootstrap testing, and some statistical results only showed moderate significance. Future investigations necessitate larger multi-center cohorts and additional datasets to robustly substantiate these findings.

## Conclusion

This study systematically elucidates the pivotal role of metabolic pathways in BCBM progression through integrated bioinformatics and multiomics approaches. Leveraging pathway activity, two different metabolic subtypes (C1/C2) with different prognostic levels were constructed, while metabolic-related gene clusters (MRGs) demonstrated superior capacity to stratify patient prognosis and immunophenotypic heterogeneity—thereby advancing precision immunotherapy strategies. The risk prediction model, anchored by hub prognostic genes (ADAM12, ALDH1A1, TLE3, PGR), has the potential to become a clinical tool. Comprehensive analyses revealed significant divergences across metabolic subtypes, MRGs, and risk groups in transcriptional programs, functional pathway enrichment, and immune infiltration landscapes. Furthermore, scRNA-seq resolved cell-type-specific expression of ALDH1A1 within TME and uncovered extensive cellular crosstalk between high/low ALDH1A1-expressing cells and cofactor/vitamin metabolic activity strata, identifying actionable metabolic-immunological targets for therapeutic intervention.

## Supporting information

S1 Table GO and KEGG analysis results. (TXT)

S2 Table Univariate analysis of clinical data. (TXT)

S3 Table Drug sensitivity analysis of different clusters. (TXT)

S1 Fig ALDH1A1 cell communication panorama. (TIF)
