# Supplementary figures and images for: Exploring the relationship between metabolism and immune microenvironment in breast cancer bone metastasis based on metabolic pathways
Source: PLoS One. 2026 Jan 29;21(1):e0341270. doi: 10.1371/journal.pone.0341270 (PMC12854414; doi:10.1371/journal.pone.0341270)

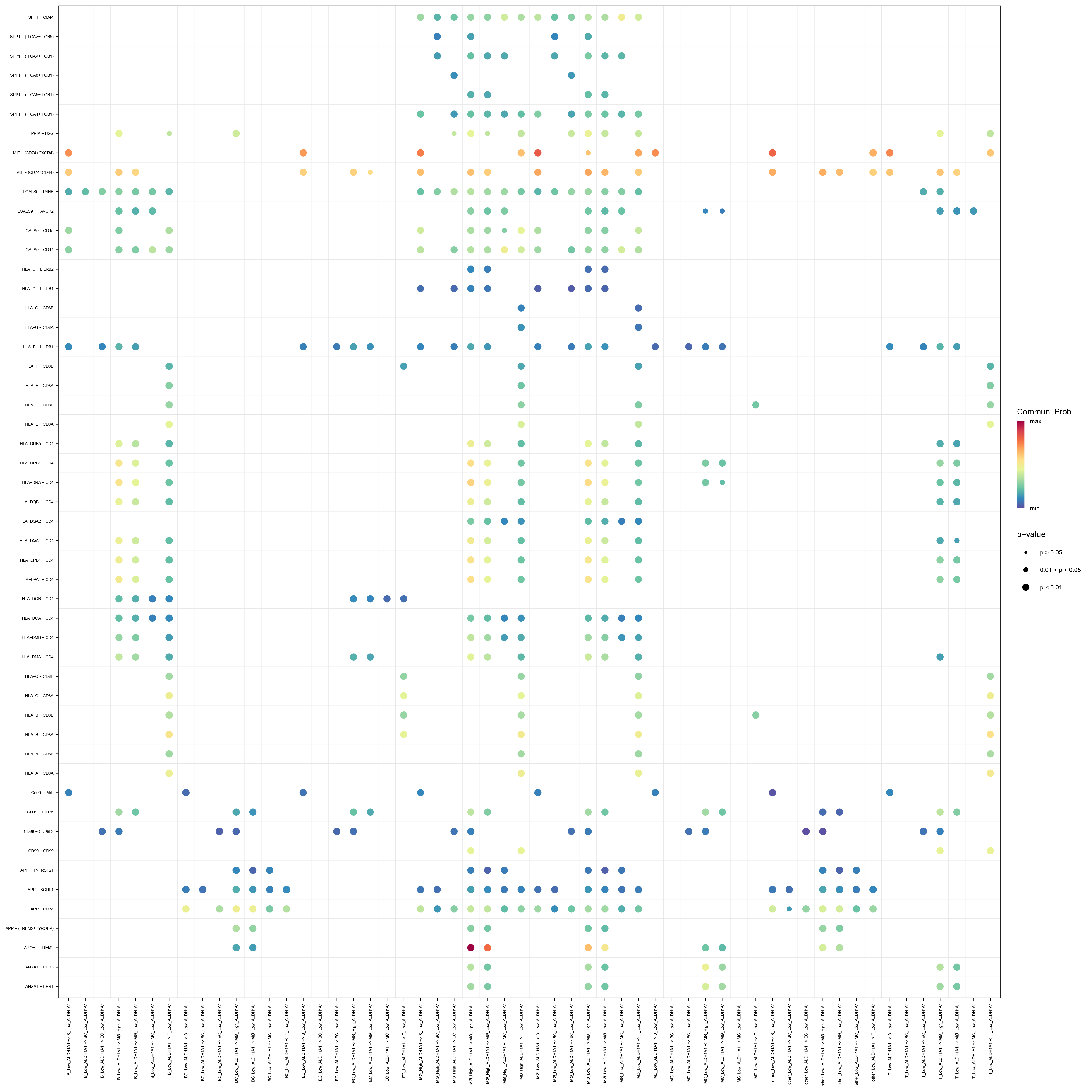

Supplement: S1 Fig — (TIF) [file pone.0341270.s004.tif]
